# A Unified Framework on Generalizability of Clinical Prediction Models

**DOI:** 10.3389/frai.2022.872720

**Published:** 2022-04-29

**Authors:** Bohua Wan, Brian Caffo, S. Swaroop Vedula

**Affiliations:** ^1^Department of Computer Science, Whiting School of Engineering, Johns Hopkins University, Baltimore, MD, United States; ^2^Department of Biostatistics, Johns Hopkins Bloomberg School of Public Health, Johns Hopkins University, Baltimore, MD, United States; ^3^Malone Center for Engineering in Healthcare, Whiting School of Engineering, Baltimore, MD, United States

**Keywords:** generalizability, external validity, clinical prediction models, explainability, prognosis, diagnosis, dataset shift

## Abstract

To be useful, clinical prediction models (CPMs) must be generalizable to patients in new settings. Evaluating generalizability of CPMs helps identify spurious relationships in data, provides insights on when they fail, and thus, improves the explainability of the CPMs. There are discontinuities in concepts related to generalizability of CPMs in the clinical research and machine learning domains. Specifically, conventional statistical reasons to explain poor generalizability such as inadequate model development for the purposes of generalizability, differences in coding of predictors and outcome between development and external datasets, measurement error, inability to measure some predictors, and missing data, all have differing and often complementary treatments, in the two domains. Much of the current machine learning literature on generalizability of CPMs is in terms of dataset shift of which several types have been described. However, little research exists to synthesize concepts in the two domains. Bridging this conceptual discontinuity in the context of CPMs can facilitate systematic development of CPMs and evaluation of their sensitivity to factors that affect generalizability. We survey generalizability and dataset shift in CPMs from both the clinical research and machine learning perspectives, and describe a unifying framework to analyze generalizability of CPMs and to explain their sensitivity to factors affecting it. Our framework leads to a set of signaling statements that can be used to characterize differences between datasets in terms of factors that affect generalizability of the CPMs.

## 1. Introduction

Clinical prediction models (CPMs), which are often referred to as risk models (Wynants et al., 2017), inform many healthcare decisions. Healthcare decisions informed by CPMs, including whether a patient has a disease (diagnosis), whether a patient will develop severe disease or other outcomes (prognosis), and whether a patient will have a certain outcome in response to a given treatment (treatment effects), are all based upon predictions given observed information. Predictions from algorithms using machine learning (ML) methods can be used to support clinical decisions, provided they have sufficient utility in some form of correctness, such as accuracy or error. However, the empirically reliable correctness of the predictions is necessary—but not sufficient—for an algorithm to be useful in practice. Among other considerations, prediction algorithms are expected to be explainable in order to be put into common use.

There is little consensus on a definition of explainability for ML methods. From a technological perspective, explainability is related to model complexity and how a prediction was computed given certain inputs (i.e., features). For example, the weights or coefficients for features from a linear model explain how the features are transformed into a prediction. A weight in a linear model can be easily interpreted as the estimated expected change in the response per unit change in the associated predictor while holding the other predictors fixed. On the other hand, deep neural networks are much less parsimonious and non-linear. In fact, they are often referred to as “black-boxes” because the specifics of how features are mapped to predictions is typically not well understood. Although some techniques, such as saliency maps, provide a *post-hoc* attempt to shed light on how a prediction was obtained from a neural network, they are not foolproof (Adebayo et al., 2018; Ghassemi et al., 2021) and can provide misleading information.

While there is broad agreement about the goals of explainability of ML methods for CPMs and predictions obtained using them, evidence on how it affects users' trust and adoption of the CPMs is limited. Explainability of predictions from ML methods may engender users' trust in them. Explanations are trustworthy and persuasive when they are intuitive and consistent with expectations given prior knowledge about a patient's clinical presentation. As such, the key purpose for obtaining explainability of predictions that inform clinical decisions is to justify their use in systems (Tonekaboni et al., 2019). Transparency, couched in terms of the specific features input in the model, allows for the validation of predictions against domain knowledge, i.e., the information used by the model to reach a prediction. In addition, information about the contexts in which the model yields inaccurate predictions enhances transparency. However, transparency in terms of certainty may decrease user's trust and adoption of the models in decision-making (Fügener et al., 2021). As such, the influence of transparency and model certainty on adoption of predictions from CPMs in decision-making is not well studied.

Another goal of explainability is to prevent the failure of a model to reproduce its accuracy in novel populations (Ghassemi et al., 2021). Failure to replicate model performance can be driven by spurious correlations in the training data that are not present in testing data or during real world usage. Correlations in the training data that represent systematic biases, for example those reflecting societal prejudices, can lead to models being biased toward or against specific subpopulations. Evaluating generalizability, or external validity, of a CPM improves its explainability through the detection of contexts in which it yields inaccurate predictions, ruling out spurious associations between features and the target (outcomes), and analyzing disparity in performance across subpopulations.

In the context of a CPM, external validity refers to evaluating it in an independent dataset, i.e., data “collected as part of an exercise separate from the development of the original model” (Royston and Altman, 2013). The external dataset is typically similar to the development dataset, and recruited either from different study sites (geographic validation) or at different points in time (temporal validation). Model performance on the external dataset is typically then evaluated in terms of discrimination and calibration. Here, discrimination refers to the extent to which the predicted probability characterizes patients with different labels (e.g., disease / no disease for diagnostic models and different risk for prognostic models) and calibration refers to the correspondence between predicted and observed risk of outcomes (Altman et al., 2009; Moons et al., 2009b).

In the ML literature, model performance on novel data is often discussed in terms of dataset shift. This refers to heterogeneity between datasets used to test the model (e.g., data from real-world settings) and train the model. In the presence of dataset shift, a model's performance often fails to generalize to a test dataset. The shifts in datasets are described with regard to the empirical distributions of the input and the target or the output (both marginal and conditional on the input).

There is discontinuity in concepts on generalizability of CPMs in the clinical research and ML literature. In the clinical research literature, failure of a CPM to generalize to a new dataset is dissected in terms of an assumed underlying population distribution (super-population), which models the data acquisition and potential biases in the study design (Hemingway et al., 2013). This approach stands on a firm theoretical foundation. However, it hinges on the connections between the superpopulation and sampling models to the data. On the other hand, in broad strokes, generalizability of CPMs in the ML literature is evaluated based on the empirical distributions of observed data. Of course, the lines between these field-specific approaches to generalizability of CPMs are blurred in much of clinical and ML research.

Despite this overlap, we argue that the discontinuity persists in the two field-specific approaches. Furthermore, this discontinuity is both artificial and wasteful, thus raising the need for an omnibus approach to generalizability of CPMs. Bridging and combining concepts from the two field-specific approaches will enable more robust algorithmic development and translation. For example, domain shift, a type of dataset shift, corresponds to both measurement error and measurement bias. Evaluating domain shift via the distribution of the predictors or outcomes alone fails to distinguish and hypothesize about the source of the error or bias. Such a mapping of domain shift to measurement error or bias allows for the application of methodology specifically designed to address the source of error or bias. For example, mismeasured covariates affect the calibration and discrimination of models (Rosella et al., 2012; Pajouheshnia et al., 2019; Luijken et al., 2020) and methods have been developed to mitigate the impact of error or bias (Khudyakov et al., 2015) in narrow settings. However, related methodological research on CPMs employing ML methods is not as well developed. Our objective is to propose a framework for unifying concepts on generalizability of CPMs with the hope of fostering further development merging the two styles of generalizability.

Our emphasis on generalizability is warranted for several reasons. From a practical perspective, CPMs support decisions in patient care. Consistent performance in heterogeneous real-world clinical settings is a key requirement for broad deployment, especially for those utilizing complex ML methods. The value of a CPM depends on how it performs in routine practice, on data not seen in training or initial model building and validation. Lack of clarity about the conditions under which a CPM does and does not fail in external validation can make it less useful to decision-makers and potentially harmful to patients. Furthermore, granular information on sources of error and bias leading to CPM failure in external validation leads to greater efficiency in the creation of new algorithms and the improvement of existing ones.

From a methodological perspective, evidence of generalizability of a CPM helps rule out the possibility of overfitting to training data, chance covariate relationships, or predictor relationships idiosyncratic to the training data. Data on false positive and false negative predictions can provide insights on when ML methods fail. Information on failure modes of CPMs promotes trust because users can anticipate when models are useful and when they are likely to mislead. Finally, evidence from the evaluation of ML methods in different subsets of patients can inform consideration of risk and outcomes in the context of patient preferences and values. Thus, importantly, addressing generalizability of ML methods can promote patient centeredness of CPMs.

The manuscript is organized as follows. Section 2 describes the problem statement for generalizability of CPMs, Section 3 explains design of studies to develop and validate CPMs, Section 4 introduces concepts on dataset shift, Section 5 proposes a unified framework for concepts on generalizability of CPMs and we conclude in Section 6.

## 2. Problem Statement for Generalizability of CPMs

### 2.1. Notation

We use *X*_*dev*_ to denote the observed predictors to CPMs in the development dataset, e.g., variables from the medical chart, images. *Y*_*dev*_ represents the observed outcome or target for prediction in the development dataset, e.g., presence of disease, severity of disease, risk of outcome. The probability distributions of these variables in the dataset used to develop the CPM (development dataset) are denoted by *P*(*X*_*dev*_) and *P*(*Y*_*dev*_). Sampling weights with which patients in the development dataset are drawn from the population are represented by *W*_*dev*_. In turn, *X*_*ext*_, *Y*_*ext*_, and *W*_*ext*_ denote the input, output, and sampling weights in the external dataset, respectively. When the subscripts are omitted, we do not make a distinction between the two datasets.

We consider probabilistic CPMs that give estimated conditional probabilities *P*_*m*_(*Y*|*X*) as output; examples include (probabilistic) graphical models and neural networks with sigmoidal output activation functions. The conditional probability output is denoted as *P*_*m*_(*Y*|*X*) for the development dataset. One could draw a slight distinction with purely functional models, which only give either class scores or direct predictions without a formal reference to a conditional probability. However, most of these models can typically be thought of as a special case of probabilistic models through a functional component of the distribution, such as a mean or argument maximum. We denote the functional component of interest as *f*(*X*). In addition, one might estimate a conditional probabilistic model without concern over calibration, i.e., that the probability is an accurate representation of real world frequencies and any monotonic function of the conditional probability would suffice. Regardless, the problem of dataset shifts impacts CPMs, whether one wants to estimate a calibrated conditional probability or simply to obtain good functional prediction estimation and both can benefit from our framework to assess the risk of dataset shifts.

### 2.2. Problem Statement for Generalizability of CPMs

We assume that a CPM is developed within the context of an underlying biologic disease process such that there is a causal association between *X* and *Y*. This assumption is consistent with clinicians' expectations that CPMs will be transparent about what features are used in the model to predict the outcome. Transparency on features allows for contrasting algorithmically important predictors and relationships with clinical judgments based on established first principles in the preclinical and clinical sciences. We also assume that the biologic process, and consequently the causal association between *X* and *Y*, may differ across the distribution of *X*. For example, the disease process may differ by patients' age. Finally, we assume that the CPM was adequately optimized to the training data.

Then, the absence of generalizability is expressed as:


$$\begin{array}{l} P_{m}\left(Y_{ext}|X_{ext}\right)\neq P\left(Y_{ext}|X_{ext}\right), \end{array}$$

where here we mean equality loosely subject to sampling and estimation variation or in terms of asymptotic convergence. A less restrictive version could be given if there is a reduced functional form of *P*_*m*_(*Y*_*ext*_|*X*_*ext*_) being estimated; then absence of generalizability is given by:


$$\begin{array}{l} f\left(X_{ext}\right)\neq g\left\{P\left(Y_{ext}|X_{ext}\right)\right\} \end{array}$$

where *g* is the function performing the summarization, such as:


$$f(X_{ext})\neq \underset{{}_{Y_{ext}\in Y_{ext}}}{\text{argmax}}P(Y_{ext}|X_{ext})$$

or


$$f(X_{ext})\neq \int_{Y_{ext}\in Y_{ext}}Y_{ext}P(Y_{ext}|X_{ext})dY_{ext}.$$

where $Y_{ext}$ denotes a set of all possible outcomes in the external dataset. In other words, absence of generalizability occurs when the estimated model does not estimate the desired quantity from the true conditional distribution in the external sample.

## 3. Concepts on Generalizability of CPMs

Generalizability of CPMs is well studied and often evaluated in the clinical research literature. The design of studies to develop CPMs and to evaluate their generalizability involves several choices that can introduce bias or otherwise limit inference about validity of the CPMs. Several articles describe the objectives and design of studies to develop and validate CPMs for diagnosis and prognosis, their reporting, and risk of bias (Altman et al., 2009; Royston et al., 2009; Whiting et al., 2011; Moons et al., 2015; Wolff et al., 2019). CPMs are developed in studies that include patients who represent a given source population. Studies on CPMs may include analyses validating them using a subset of the development dataset, i.e., internal validation, or using an external dataset obtained from a new sample of patients, i.e., external validation, or both (Wolff et al., 2019).

Multiple terms related to external validity are described in the clinical research literature (Justice et al., 1999). Validity of a CPM in a new sample of patients drawn from an identical source population as that in the development study is often termed as reproducibility. On the other hand, validity of a CPM in a new sample of patients drawn from different—but plausibly related—population or using different methods to collect data compared with the development study is referred to as transportability. In turn, there are different types of transportability, including historical (validity in samples from different point in calendar time), geographical (validity in samples from different locations on the globe), methodologic (validity when data are collected using different methods), spectrum (validity in patients with different degrees of disease severity), and follow-up (validity of CPMs for prognosis in patients who were followed up for different durations).

Analysis of generalizability of CPMs includes calibration and discrimination. Calibration refers to the agreement between predicted probabilities and their underlying estimands. For example, if the CPM assigns an average probability of 0.4 for a subset of patients, then 40% of the relevant population in that subset should actually have the outcome. Of course, population calibration would be evidenced by relevant sample calibration. It should be emphasized that many standard validation metrics do not consider calibration. For example, non-parametrically estimated receiver operating characteristic curves only depend on the ranks of the predictions, thus can have no relevant calibration information. Some methods to analyze calibration are described in Copas (1983), Spiegelhalter (1986), and Steyerberg (2009). Calibration is primarily a concern in CPMs if physicians or users will be relying the actual values of the estimated probability. A good example would be comparing CPM output with traditional estimates of risk. If the CPM is not calibrated, such comparisons can not be made, regardless of how well the algorithm performs at discrimination. To elaborate, discrimination refers to the ability of the CPM to separate patients with and without the outcome. A common measure of discrimination is the C-statistic, which corresponds to the area under the receiver operating characteristic curve (Spiegelhalter, 1986). Other important statistical issues about studies on generalizability of CPMs include sample size, which are discussed in other sources (Riley et al., 2020).

It is well known that the design of studies to develop CPMs can introduce biases or errors in their estimated measures of validity (Whiting et al., 2011; Damen et al., 2019; Wolff et al., 2019). Studies on generalizability of CPMs are susceptible to bias in a similar manner. Studies on generalizability include patients who meet prespecified eligibility criteria, which determine their similarity to those included in the sample used to develop the CPM. Of note, duration of follow-up is an important eligibility criterion, because it allows for the study of follow-up transportability (Justice et al., 1999). In addition, predictors input to the CPM and its outputs (i.e., outcomes) should be completely measured in all patients included in the generalizability study using processes that do not introduce any more error than that in the original development study. For predictors and outcomes with multiple dimensions, e.g., time series, images, or videos, it is necessary to measure them using processes that correspond to those used in development of CPMs. Error and bias in measuring predictors used in CPMs adversely affect calibration and discrimination (Rosella et al., 2012; Khudyakov et al., 2015; Luijken et al., 2019, 2020; Pajouheshnia et al., 2019). In another instance, in generalizability studies on CPMs for diagnosis, ascertaining the outcome using information on the predictors can lead to over optimistic estimates of validity, i.e., incorporation bias (Whiting et al., 2011).

Studies to develop CPMs using machine learning methods often ignore potential for bias from various aspects of their design. For example, only a few among 62 studies developing CPMs for diagnosis or prognosis of coronavirus disease 2019 (Covid-19) were assessed to be at low risk of bias based on study design (Roberts et al., 2021). Similar findings were reported in a comprehensive systematic review of studies developing CPMs for diagnosis or prognosis of Covid-19 using any data source (Wynants et al., 2020). This deficit in rigor in study design may be because well-known concepts on potential sources of biases from design of studies to develop and evaluate CPMs are not well disseminated to the ML researchers.

## 4. Dataset Shifts

In the context of CPMs, we consider dataset shifts as the differences in the distributions between development and external data. The patterns and types of dataset shifts have been exhaustively described in the ML literature. Storkey has cataloged the common types of dataset shifts according to their causes (Storkey, 2008). Subsequently, Moreno-Torres, et.al., categorized dataset shifts into four groups, namely covariate shifts, prior probability shifts, concepts shifts, and other types of dataset shifts (Moreno-Torres et al., 2012). Finally, Kull and Flach extended the ideas to provide an exhaustive listing of dataset shifts (Kull and Flach, 2014).

To simplify the discussion on dataset shifts and enable readers from different scientific domains to identify common concepts, we consider three basic types of dataset shift: covariate shift, prior probability shift, and concept shift. Other types of dataset shift described in the literature result in one or more of the basic types of dataset shift listed above. We adopt previously described definitions of the dataset shifts; however, we use selection diagrams to illustrate them. As noted in Box 1, selection diagrams are a well developed, succinct, and unifying way to denote distributional and structural differences between datasets, in addition to allowing reproducible analysis of transportability in both randomized and non-randomized contexts (Pearl and Bareinboim, 2011). Furthermore, using selection diagrams helps establish a standard way to describe dataset shifts and facilitate the discussion, because custom graphical diagrams were devised in previous works to depict them (Storkey, 2008; Moreno-Torres et al., 2012; Kull and Flach, 2014).

Box 1 **Terminologies**

**Table:** 

1.	Causal graph or causal diagram (Pearl, 1995): Directed Acyclic Graphs that illustrate conditional independent associations and qualitative causal influences. By convention, the solid circles denote observable variables such as X, W and Y. The unobservable variables such as measurement error, *U*_*x*_, are denoted as hollow circles. The solid arrows represent causal associations.	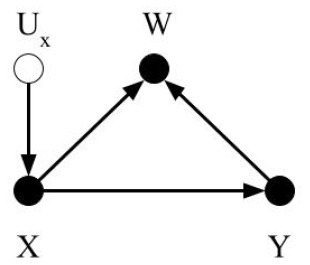
2.	Transportability (Pearl and Bareinboim, 2011): Given two domains Π_*dev*_ and Π_*ext*_, such as a development dataset and an external dataset, characterized by probability distributions *P*_*ext*_ and *P*_*dev*_, and causal diagrams $G_{dev}$ and $G_{ext}$, respectively, a statistical relation *R* is said to be observationally transportable from Π_*dev*_ to Π_*ext*_ over *V*_*ext*_, a subset of variables, if *R*(*P*_*ext*_) is identified from $P\text{, }P_{ext}(V_{ext})\text{, }G_{dev}\text{, and }G_{ext}\text{, where }P_{ext}(V_{ext})$ is the marginal distribution of *P*_*ext*_ over *V*_*ext*_. Transportability is helpful to decide whether a learned relationship can be applied in new data.	
3.	Trivial Transportability (Pearl and Bareinboim, 2011): The relationship *R* can be estimated given the causal diagram, $G_{ext}$, and full probability distributions, *P*_*ext*_.	
4.	Selection diagram (Pearl and Bareinboim, 2011): Selection diagrams are augmented causal diagrams. Selection diagrams contain an extra set of “selection variables”, *S*. Selection variables correspond to mechanisms by which the variables to which they point differ between the development dataset and the external dataset. For example, in the selection diagram example figure on the right, the variable *W* is pointed by the selection variable *S*, which means *W* will be different between the development dataset and the external dataset.	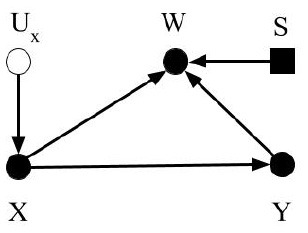
	These selection variables are denoted by solid rectangles. The selection diagram is a succinct and unifying way to identify causal associations that can transport from the development dataset to the external datasets, and to denote distributional and structural differences between the development dataset and external datasets.	
5.	D-separated (Pearl, 1995): Let *A*, *B*, and *C* be three disjoint subsets of nodes in a directed acyclic graph G, and let *p* be any path between a node in *A* and a node in *B*. The path *p* here means any successive edges, regardless of their directions. *C* is said to block *p* if there is a node *w* on *p* satisfying one of the following two conditions: 1. *w* has converging arrows along *p* and neither *w* nor any of its descendants are in *C*, or 2. *w* does not have converging arrows along *p* and *w* is in *C*.	
6.	S-admissibility (Pearl and Bareinboim, 2011): Let D be the selection diagram and *S* be the set of selection variables. The *Z*-specific relationship *P*(*Y*|*X, Z*) is transportable from the development dataset to external dataset if *Z* d-separates *Y* from *S* in the *X*-manipulated version of D denoted by $D_{\bar{X}}$. In $D_{\bar{X}}$, nodes in *X* are excluded. Then, *Y*⊥⊥*S*|*Z* in $D_{\bar{X}}$. The set of variables in *Z* are called *S*-admissible.	

Our discussion of dataset shifts differs from previous descriptions in a few ways. First, the three basic types of shifts are not considered as mutually exclusive or isolated, unlike other treatments. For example, covariate shift was previously defined as a shift that causes *P*(*X*) to change whereas *P*(*Y*|*X*) remains unchanged (Storkey, 2008; Moreno-Torres et al., 2012; Kull and Flach, 2014). In contrast, our definition omits the requirement that *P*(*Y*|*X*) should remain unchanged under covariate shift. In addition, we consider the pragmatic scenario that more than one dataset shift may be simultaneously observed. Finally, we view the three basic dataset shift types as the fundamental core elements of dataset shift with the other types being a cause or a combination of the three basic types. Box 1 shows terminologies used in the rest of the discussion in this section.

### 4.1. Three Basic Types of Dataset Shift

#### 4.1.1. Covariate Shift

**Figure 1:**
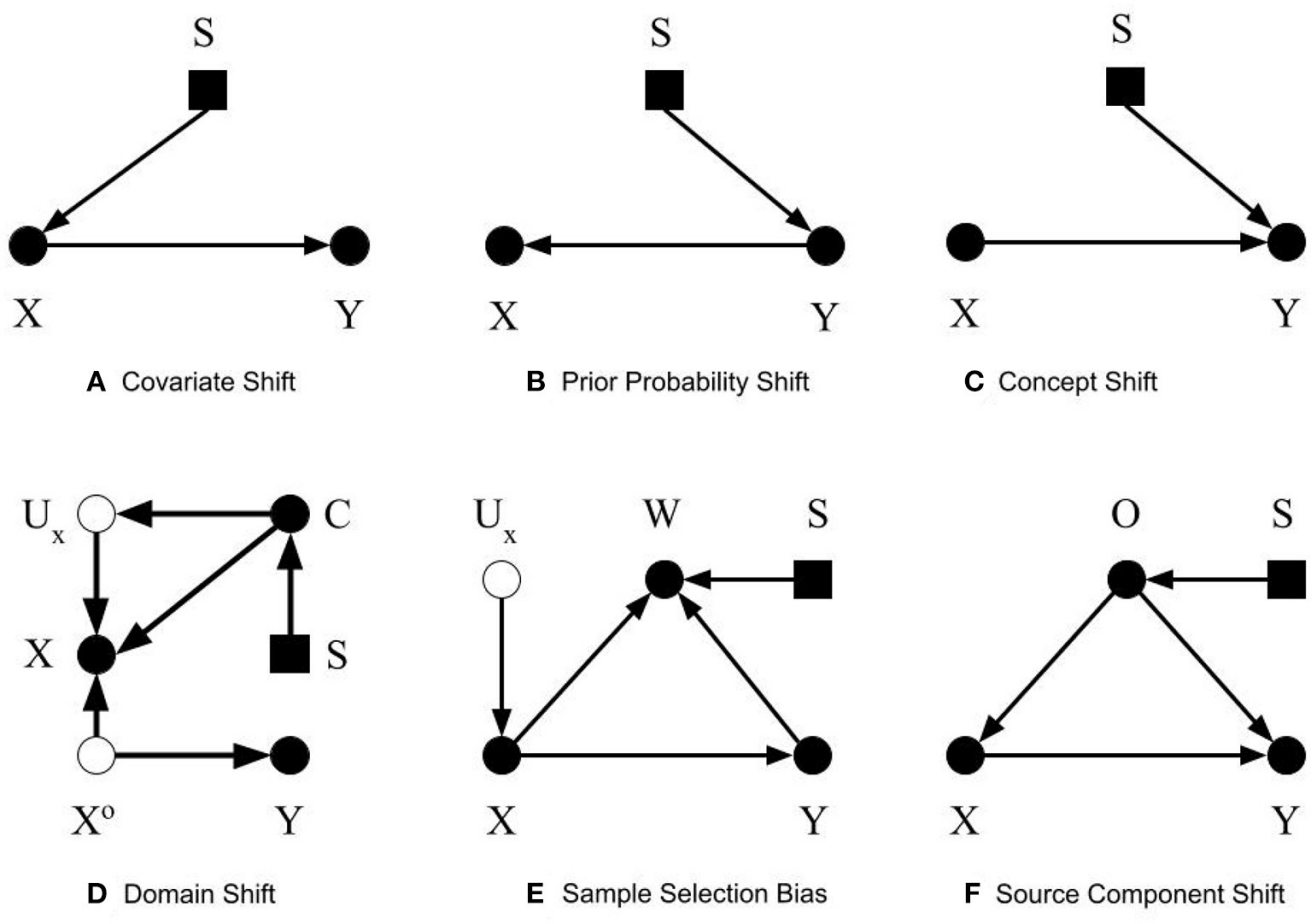
Selection diagrams for dataset shifts. The solid circles denote observable variables. The hollow circles represent unobservable variables. The rectangles denote the selection variables.

We define covariate shift as the differences in the distribution of covariates between datasets, e.g., the development and external datasets. The covariate is the input to the CPMs, or the predictors, which can be symptoms or other variables from the medical chart, images, time series data, etc. Covariates are named as such because they are presumed to “co-vary” with the outcome, i.e., their variation is related to outcome variation. For this discussion, covariate shift only refers to differences in the distribution; the covariates themselves and their dimension is assumed to be identical between the datasets. Formally, covariate shift is observed when *P*(*X*_*dev*_)≠*P*(*X*_*ext*_). As mentioned, most previous work (Storkey, 2008; Moreno-Torres et al., 2012; Kull and Flach, 2014) assumes that *P*(*Y*_*dev*_|*X*_*dev*_) = *P*(*Y*_*ext*_|*X*_*ext*_) to isolate the discussion of covariate shift. We suggest omitting this requirement, allowing the conditional relationship to depend on the covariate distribution itself. That is, in the context of CPMs, not only has the distribution of demographic and health characteristics in patient population changed, but also their relationship to the outcome. The easiest way this can occur is if there is an unmeasured unknown predictor that impacts both the covariates and the outcome. An example in our own research contrasted sleep measurement in the general population versus sleep measurement in patients referred sleep clinics to diagnose sleep apnea. Subselecting a group in the general population that matches the clinical population on measured characteristics does not provide equivalent prediction performance. Why? Because the entirety of factors that cause a patient to be referred to a sleep clinic are not measured (Caffo et al., 2010). The selection diagram shown in Figure 1A illustrates our definition. The selection variable *S* points to the variable *X* denoting the differences in distribution between the two datasets.

A common example of covariate shift in CPMs is when a model developed using data from adults is evaluated in children (Moons et al., 2009a). In addition, covariate shift may arise from the inclusion of different patient subpopulations (e.g., patients of different ages), measurement processes (e.g., spiral computed tomography vs. conventional computed tomography), data preprocessing (Um et al., 2019), or incomplete measurement of the predictors or the outcome (e.g., secondary to change in testing policy Singh et al., 2019; Subbaswamy et al., 2021). Covariate shift is particularly concerning when a nonlinear function maps the predictors to the output (Altman and Bland, 1998). Furthermore, covariate shift in terms of some predictors, such as sex, gender, ethnicity, race, or age raises concerns about the fairness of CPMs.

Covariate shift can be avoided if the external dataset is well designed; that is, the external dataset encapsulates the distribution of covariates in the development dataset where the learned causal relationship between *X* and *Y* is valid. Then, one can directly transport the relationship between *X* and *Y* to the external dataset. This transportability is evident because *Y* is conditionally independent of *S* given *X*.

#### 4.1.2. Prior Probability Shift

Prior probability shift is defined similarly to covariate shift. Prior probability shift refers to the differences in the prior distribution of the outcome between the development dataset and the external dataset. In probabilistic terms, if the *P*(*Y*_*dev*_)≠*P*(*Y*_*ext*_), then a prior probability shift is observed. In biostatistical parlance, prior probability shift occurs if the disease prevalence shifts. If the outcome represents a disease, then prior probability shift corresponds to a difference in the disease prevalence between the training and evaluation datasets. In previous work (Storkey, 2008; Moreno-Torres et al., 2012; Kull and Flach, 2014), an additional condition for prior probability shift was that *P*(*Y*_*dev*_|*X*_*dev*_) = *P*(*Y*_*ext*_|*X*_*ext*_). We omit this requirement and consider the pragmatic scenario in which prior probability shift may be caused by other types of dataset shifts. Figure 1B shows the selection diagram for prior probability shift. The selection variable now points to variable *Y* denoting the differences in the distribution of *Y* between the two datasets.

Prior probability shift is also very common in the context of CPMs. For example, when a CPM to diagnose deep vein thrombosis developed using data from a secondary care setting was evaluated in patients from a primary care setting, it showed lower sensitivity and specificity (Wells et al., 1997; Oudega et al., 2005). This adverse effect on model performance is not surprising, because patients in a secondary care setting are a select subset of patients seen in primary care who are more likely to have the target condition. Another common context for prior probability shift is a difference between datasets in the duration for which patients were followed up when evaluating CPMs for prognosis. The natural progression of disease means that patients who were followed up for different times are at differing risks of outcome.

The causal relationship between *Y* and *X* is transportable, as *X* is conditionally independent to *S* given *Y*. Consequently, if an accurate estimate of *P*(*Y*_*ext*_) is available, then we can simply use *P*(*Y*_*ext*_) instead in the model to address prior probability shifts. The conventional method to address prior probability shift is to adjust the intercept in the external dataset (Moons et al., 2012). However, when the CPM is used in clinical practice, it is hard to obtain an accurate estimate of *P*(*Y*_*ext*_).

#### 4.1.3. Concept Shift

Concept shift is defined as the difference in the conditional distribution of *Y* given *X* between the development dataset and the external dataset. Concept shift is observed when *P*(*Y*_*dev*_|*X*_*dev*_)≠*P*(*Y*_*ext*_|*X*_*ext*_). This definition is different from that in previous works (Storkey, 2008; Moreno-Torres et al., 2012; Kull and Flach, 2014), since we excluded their requirement that *P*(*X*_*dev*_) = *P*(*X*_*ext*_). Additionally, we do not consider the dual problem where the causal relationship is reversed from *X* causing *Y* to *Y* causing *X*, because it is less pertinent to CPMs (Section 2.2). Figure 1C shows the selection diagram for concept shift. Both the selection variable *S* and *X* point to variable *Y*. It denotes that the relationship from *X* to *Y* differs between datasets.

Concept shift is hard to identify, and even harder to mitigate. Concept shift ensues when the underlying biology or causal associations between the predictors and the outcome differ. Some reasons for such differences include distinct patient subpopulations, and changes in measurement of predictors or outcomes. Conventional approaches to address failure of CPMs due to concept shift include updating coefficients for predictors, and adding new predictors to improve CPM performance in the external dataset (Moons et al., 2012). However, a difference in the underlying biology is not necessarily detectable from the observed data; clinical knowledge is necessary.

In the presence of concept shift, the relationship between *X* and *Y* is only trivially transportable, which means that the external dataset needs to be fully observed and *P*(*Y*_*ext*_|*X*_*ext*_) should be re-learned. This requires knowing the full probability distributions of Y* and X*. Therefore, trivial transportability is of little use. Importantly, concept shift is disproportionately impacted in highly non-linear models, like neural networks and other machine learning approaches. For example, a simple regression relationship, averaging over non-linearities and interactions, may continue to hold (thus be transportable), even if concept shift has occurred. Thus, in the present of concept shift, summaries of an accurate CPM model may continue to apply in transport, even if the model itself does not.

### 4.2. Causes of Dataset Shifts

In this subsection, we describe a few causes of dataset shifts introduced in Storkey (2008). Since any dataset shift involves differences in the predictor distribution, the outcome distribution, or the concept distribution, it can be described as a cause of at least one of the three basic types of dataset shifts introduced in the previous subsection.

#### 4.2.1. Domain Shift

Domain shift is caused by changes in the measurement processes, or the representation of the predictors, outcomes, or both. These changes result in differences in the measures themselves, or the type and magnitude of measurement error or both. When changes in predictor distribution are not accompanied by corresponding changes in the outcome, concept shift will be observed. Differences in measurement or description of the outcome results in prior probability shift. Figure 1D shows the selection diagram for domain shift. *C* denotes the measurement unit, e.g., device used to capture images. *U*_*x*_ represents the error of measurement, which is typically unobserved. *X*^*o*^ is the true value of the predictors. This selection diagram only shows the case when predictors, but not the outcome, are subject to measurement bias. Differences between datasets in the measurement units, for example, imaging systems from different manufacturers or the proficiency of clinicians performing an exam, introduce domain shift.

The true value of predictors or outcomes are often hard to obtain. Any predictor or outcome measured for use in CPMs is subject to measurement error. Hernán and Cole (2009) explain four types of error in measurement: (1) independent and non-differential, (2) independent and differential, (3) dependent and non-differential, (4) dependent and differential. To elaborate, measurement error in the predictors is independent when it is not affected by error in measuring the outcome. Measurement error in the predictors is non-differential when it does not differ across the distribution or levels of the outcome. The presence of measurement error implies that the observed relationships between the measured predictors and the measured outcomes might not not reflect the actual causal relationship between *X*^*o*^ and *Y*.

In the selection diagram shown in Figure 1, *X*^*o*^ is d-separated from *S*, so the causal relationship between *X*^*o*^ and *Y* is transportable. Furthermore, since *C* is S-admissible, the association between *X* and *Y* is also transportable given observations of *C*_*ext*_, as long as the association between *C* and *U*_*x*_, and *U*_*x*_ and *X* can be estimated and they are not changing between the development and external datasets.

#### 4.2.2. Sample Selection Bias

Sample selection bias describes the type of dataset shifts that are caused by sampling weights. The joint distribution of *X* and *Y* is dependent on the sampling weights. Formally, the joint probability of a sample will be *P*(*X, Y*) = *P*(*X, Y, W* = 1) = *P*(*W* = 1|*X, Y*)*P*(*Y*|*X*)*P*(*X*). A sample is only selected when *W* = 1. As stated in Moreno-Torres et al. (2012), three basic patterns of dataset shifts may occur because of sample selection bias: (1) covariate shift, when *P*(*W* = 1|*X, Y*) = *P*(*W* = 1|*X*), (2) prior probability shift, when *P*(*W* = 1|*X, Y*) = *P*(*W* = 1|*Y*), and (3) concept shift when *W* is dependent on *P*(*Y*|*X*). The selection diagram shown in Figure 1E describes sample selection bias. The selection variable *S* points to the sampling weights *W*. Since *S*, the predictors *X*, and the outcome *Y* points to *W*, the dependency from *Y* to *W*, *X* to *W*, and *W* itself could be different between datasets.

Many mechanisms that introduce selection bias in epidemiologic studies are relevant to understand sample selection bias and its influence on generalizability of CPMs (Hernán et al., 2004). Sample selection bias affects generalizability of CPMs, because the true relationships among *X* and *Y* in the target population are not reflected in the study sample. A common scenario for sample selection bias is to selectively recruit patients who may be less or more sick than other patients. For example, CPMs developed using data from patients admitted to hospitals, who are sicker than those in an outpatient setting, may not generalize to patients in an outpatient setting. Another common scenario for sample selection bias arises from missing data from a select subset of patients, because of differential losses to follow-up or mechanisms to capture data, self-selection of patients volunteering to participate, etc.

The *W* variable in the selection diagram shown in figure is S-admissible, which means should we obtain the *W*_*ext*_ and its conditional relationship with *X*_*ext*_ and *Y*_*ext*_, we can estimate the conditional relationship between *X*_*ext*_ and *Y*_*ext*_.

#### 4.2.3. Source Component Shift

Source component shift is another common reason for dataset shift (Storkey, 2008; Kull and Flach, 2014). Usually, data are generated from a variety of sources. Source component shift happens when the contributions from the sources differs between the external dataset and development dataset. Source component shift can be categorized into three subtypes:

- Mixture component shift, which is caused by a change in the portion of data generated by each source. The original source of each datum is unknown. Prior probability shift and covariate shift may occur when the distribution of the prior and covariates differ among different sources. Concept shift may also be induced if the sources evolve with time. For example, consider a prognosis model to predict outcomes from Covid-19 based on symptoms and imaging at the time of hospital admission. The relationships among symptoms, imaging, and disease severity evolve over time for reasons such as the population being vaccinated, new variants of the virus, etc.
- Mixing component shift, which is similar to mixture component shift, except that each datum is averaged or aggregated from that of each source. Therefore, only mean values or the aggregated values are observed;
- Factor component shift, which results from differences between the development and the external datasets in the weights for each component when the data can be factorized into several components. One example is when the data are generated from a mixture of Gaussians. The weights of each Gaussian could be different between datasets and cause the distribution of the data to shift.

The selection diagram shown in Figure 1F describes the source component shift. The variable *O* denotes the source, which has a causal relationship with *X* and *Y*. The selection variable *S* points to *O* shows that *O* could be different between the datasets. The causal relationship between *X* and *Y* is not transportable as both *X* and *Y* are dependent on the selection variable *S*.

## 5. Unified Framework for Dataset Shifts for CPMs

We discuss generalizability of a CPM between the development dataset (i.e., dataset used to develop the CPM) and an external dataset (used to test the CPM). However, our discussion is also applicable when evaluating a previously developed CPM on two or more external datasets. Shifts are manifest differences between datasets that satisfy two conditions: (1) the differences must not be caused by sampling variability; and (2) the observed patterns of differences that distinguish the datasets from each other should be systematic. Typically, one emphasizes the patterns of differences, but not the underlying mechanisms that cause the differences. This is because multiple systematic or random mechanisms may cause an observed pattern of differences between datasets. For example, non-differential measurement error may still cause systematic differences if it is dependent upon the outcome.

Consider a CPM developed using a very large dataset that uniformly represents the full distribution of the predictors and outcomes that are measured without error. When the CPM is tested on a dataset drawn from the same population as the development dataset, with identical sampling weights, then there is no dataset shift and any difference in performance of the CPM between the two datasets depends upon the algorithm's theory (e.g., model complexity). Note that we are distinguishing between random empirical differences in the dataset and differences in the generating process and mechanisms. Specifically, we do not consider realized empirical shifts in the distribution, despite identical data generating processes and mechanisms as dataset shift.

**Figure 2:**
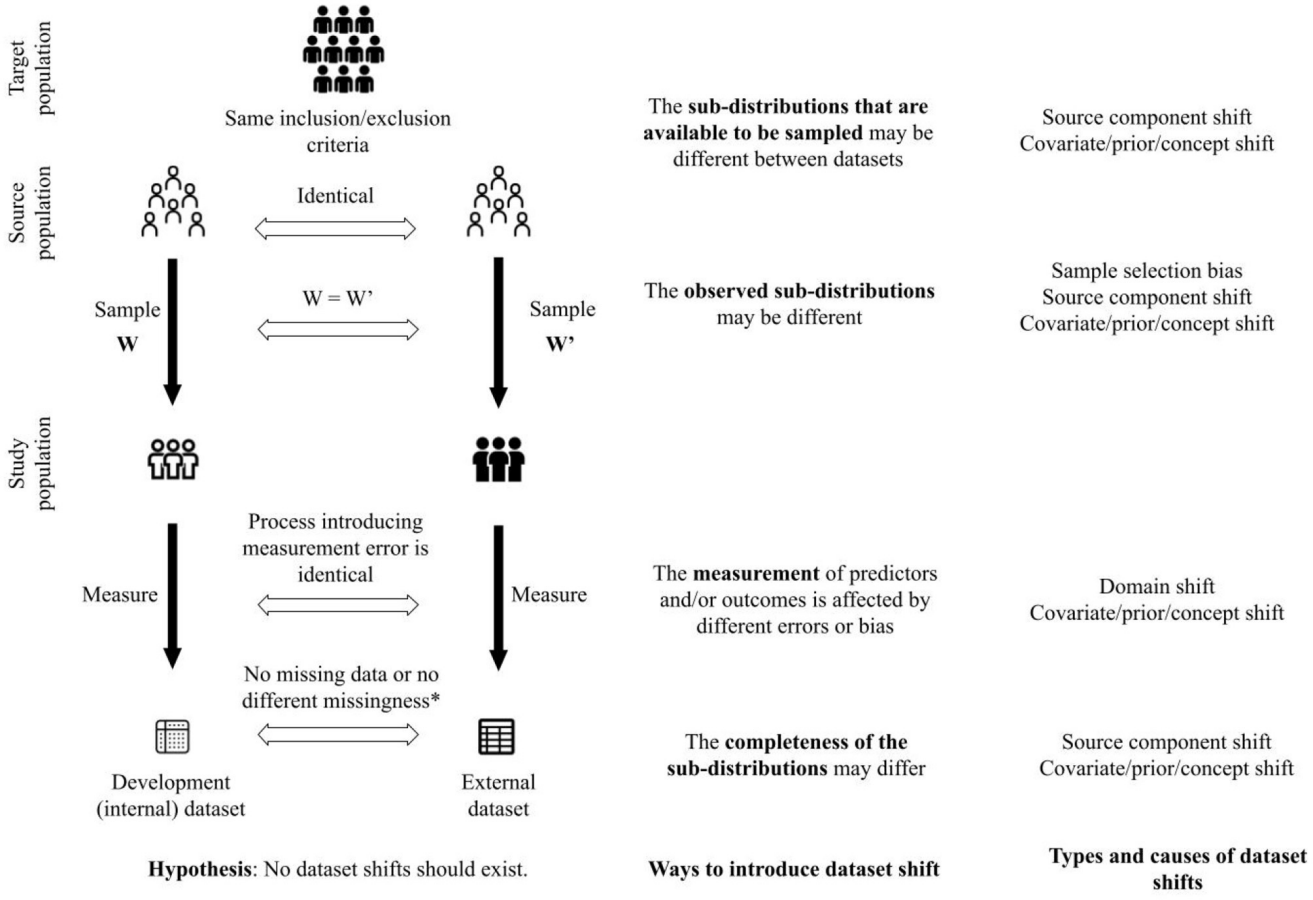
A framework to unify concepts related to generalizability of clinical prediction models. *This criterion is satisfied when there are no missing data in the development and external datasets. Furthermore, when there are missing data, there is no difference in assumptions about the missingness between the datasets (e.g., missing completely at random in both datasets), or there is no difference between the process that introduced missingness in each dataset.

To elaborate, as shown in Figure 2, we hypothesize that no shifts are expected between datasets when patients are sampled from the same source population with identical sampling weights, the predictors and the outcomes are measured using the same processes, and there are no missing data, or in the presence of missing data, there are no difference in types of missingness and processes introducing missingness. To sample patients from the same source population, the studies yielding the datasets should have the same eligibility criteria to include and exclude patients. These criteria pertain not only to patient characteristics but also to criteria such as duration of follow-up, feasibility of outcome measurement, etc.

However, no shift between datasets does not necessarily mean identical measures of calibration or discrimination for a CPM evaluated on them; model performance may still differ because of random sampling variability or error. That is, we distinguish classical sampling error and dataset shift. Consider a relatively simple example. We simulated training and evaluation of a CPM using three different methods including linear regression, polynomial regression, and a multi-layer perceptron. Parameters to replicate our simulation are shown in Table 1. To generate sample datasets, we used a mixture of Gaussians as the distribution of the covariate. For the linear regression model, we used a random linear model as the posterior. For both the polynomial regression model and the multi-layer perceptron, we used random polynomial models as the posteriors. Given a distribution of the covariate and a posterior, we uniformly sampled a training dataset of 10,000 patients and 1,000 test datasets of different sample sizes shown in Table 1. We trained each model and computed the mean squared error (MSE) using each test dataset. Using each set of MSEs from 1,000 test datasets, we plot a Gaussian with their mean and standard deviation and a histogram of their density. We expect the distribution of MSEs from the 1,000 test datasets will be normal, per the central limit theorem, because the data are independently and identically drawn from the same source and no dataset shifts were introduced.

**Table 1:** Hyper-parameters used in the simulation experiment.

**Simulation**	**Mechanism**	**Hyper-parameters**	**Value**
Covariate distribution	Mixture of Gaussian	# of components	10
		Mean	(–5,5)†
		Variance	(0,1)†
Posterior process	Random linear model (*f*_(*x*)_ = *ax*+*b*)	a	(–2,2)†
		b	(–2,2)†
	Random polynomial model	Degree	5
		Root range	(–5,5)†
Models	Linear regression model	n - o hyper-parameters	
	Polynomial regression model	Degree	5
	Multi-layer perceptron	Hidden layers	[100,100]
		Activation function	ReLU
		Optimizer	Adam
		Maximum iteration for training	1000

*Every variable is subject to a noise sampled from N(0,1)*.

*† The value is sampled uniformly from the range. ReLU, Rectified Linear Unit*.

**Figure 3:**
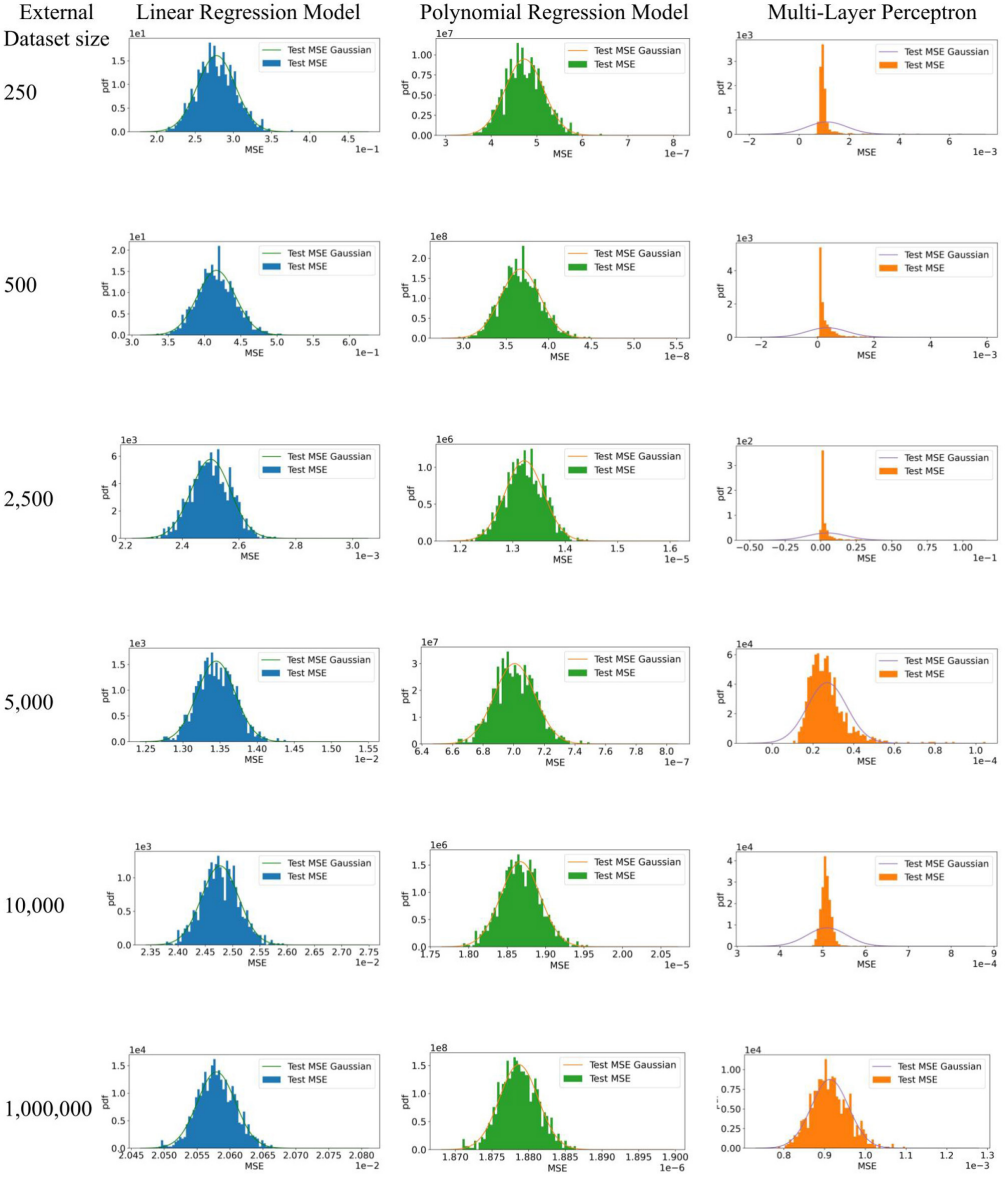
Simulation to illustrate model performance in external datasets with no dataset shifts. 1. The expectation of the estimate of algorithm performance in a test dataset is the mean of a distribution of estimates obtained by evaluating the algorithm on multiple test datasets. In other words, a difference in the magnitude of the error in the test and development datasets does not necessarily indicate poor or better algorithm performance. 95% confidence intervals of the estimate in a test dataset, which indicate the width of true distribution of estimates, are necessary. 2. Test datasets of sufficient sample size are necessary to minimize bias in the estimate of algorithm performance, depending on model complexity.

Figure 3 shows expected behavior of estimates of performance of CPMs when there are no challenges to their generalizability. Each set of MSEs necessarily followed a normal distribution as the testing data was large enough for the MSEs from the MLP to converge. It is well known that test datasets of sufficient sample size are necessary to minimize bias in the estimate of algorithm performance, depending on model complexity. In practice, CPMs are evaluated in a few test datasets, unlike the 1,000 test datasets used in our simulation. Differences in magnitude of prediction error between a few test datasets does not necessarily indicate better or worse performance of the CPM. Appropriate measures of uncertainty and variation, such as employing confidence intervals, are necessary to compare empirical MSEs. Finally, the absence of dataset shifts does not guarantee generalizability of a CPM. The size of the training dataset can influence both whether there is appropriate information to fit complex models, and without proper controls, the extent of overfitting. In these cases, purely empirical reasons can result in poor practical generalization to testing datasets, even when the testing and training sets are sampled from the same source without any form of dataset shifts.

Given our hypothesis (illustrated in Figure 2), and in the simulation described earlier in this section, we define the full distribution as the distribution that characterizes the joint probability of predictors and outcomes in the target population. The target population is a super-population, i.e. an unobserved population distribution, in which the CPMs are intended to be used. However, datasets are created from a subset of the target population defined by the inclusion and exclusion criteria and sampling weights in studies to develop and validate CPMs. We refer to the conditional distribution that characterizes the joint probability of predictors and outcomes in the subset of the target population as a sub-distribution (i.e., distribution of a sub-population). We suggest that varying the following components in the process of creating datasets (shown in Figure 2) can introduce shifts between datasets (development vs. external datasets or among different external datasets) and challenge generalizability of CPMs:

1. the sub-distributions that are available to be sampled;
2. the observed sub-distributions;
3. the measurement of predictors and/or outcomes;
4. the completeness of the observed sub-distributions.

Our framework in Figure 2 shows a proposed approach to unify dataset shifts in the context of CPMs. Other details such as model specification are beyond the scope of this narrative. Furthermore, we consider the models to be correctly specified and the challenge to generalization of the CPMs is limited to dataset shifts.

The four ways to introduce dataset shift shown in Figure 2 correspond to the process by which datasets are created. CPMs are meant to be used in a target population of patients. The eligibility criteria to include and exclude patients from whom data are collected define the source population, i.e., the subset of the target population that is available to be sampled to create the study population. Different source populations between datasets introduce source component shift. The weights used to sample patients from the source population define the study population. Different sampling weights between datasets introduce sample selection bias and source component shift. Predictors and outcomes are measured in the study population. Different processes of measuring the predictors and outcomes introduce domain shift. Finally, the measured predictors and outcomes may be incomplete, e.g., through missing data or inability to measure certain predictors or outcomes. Different patterns of incompleteness of data introduce source component shift.

The four ways to introduce dataset shift described above are neither mutually exclusive nor tend to occur independently. In fact, we conjecture that they co-occur more often than not. For example, datasets collected from patient samples defined by different eligibility criteria, i.e., source component shift, may cause a covariate shift, prior probability shift, and concept shift. In another instance, completeness of observations, as well as measurement, may be associated with which sub-distributions are observed (i.e., dependent measurement error and missingness at random). The interrelated nature of how dataset shifts may be introduced means that a certain type of shift observed between datasets may have been introduced in more than one way. Investigation into the source of the dataset shift should address all the ways in which it can be introduced. For example, a covariate shift in the predictors may be because of differences in how they are measured, or because patients from a different spectrum of disease severity were sampled using new eligibility criteria. In addition, if the process of creating two datasets differs in a certain way (e.g., different measurement processes), then the datasets should be investigated for more than one type of shift. Finally, absence of empirical differences in distributions of predictors or outcomes between datasets should not be misinterpreted as absence of dataset shift as we have defined it. It is necessary to characterize the datasets in terms of all four ways in which datasets shifts can be introduced in order to understand potential for the shifts.

Table 2 shows signaling statements to characterize a dataset relative to another dataset. The signaling statements correspond to the four ways to introduce dataset shifts shown in Figure 2. Of note, the statements on measurement and completeness apply to each predictor and outcome in the dataset. The evaluation for each statement may be described either as a binary response or as agreement or disagreement on any Likert scale. While we do not advocate any one evaluation approach, this choice may be guided by evidence on inter-rater reliability from future research.

**Table 2:** Signaling statements to characterize datasets for generalizability of clinical prediction models.

**Ways to introduce dataset shifts**	**Causes of dataset shifts**	**Signaling statements to evaluate potential for shifts between different datasets**
The sub-distributions that are available to be sampled are different	Source component shift	• The process for selecting patients from the target population into the source population for the datasets relied upon the same inclusion and exclusion criteria.
The observed sub-distributions are different	Sample selection bias Source component shift	• The process for selecting patients into the datasets (sampling weights) resulted in different proportions of sub-populations from a similar source population. • The association between predictors and the outcomes is likely to differ between the sub-populations defined in terms of a variable used for sampling patients from the source population.
The errors or biases affecting the measurement of predictors and/or outcomes are different	Domain shift	• The processes introducing error into measurement of the predictors in each dataset are identical. • The processes introducing error into measurement of the outcomes in each dataset are identical. • The definition of the outcomes in each dataset does not include information from the predictors.
The completeness of the observed sub-distributions is different	Source component shift	• Each predictor and outcome in the datasets is either a complete observation (i.e., no missingness) or it is incomplete with missingness completely at random. • One or more of the same predictors or the same outcomes in the datasets are incomplete with missingness at random. • One or more of the same predictors or the same outcomes in the datasets are incomplete with missingness not at random. • The process introducing missingness into each predictor or outcome with missing data is identical among the datasets.

*All three basic types of dataset shifts, i.e., covariate shift, prior probability shift, and concept shift, may result from each way to introduce dataset shifts shown in this table*.

Explaining the causes of the dataset shifts in the terms of the source of the data and the data generating mechanisms and processes allows for improving the explainability of CPMs. Understanding how datasets are different from each other is critical to detect and explain failure to reproduce or transport, i.e., generalize CPMs. Analysis of sensitivity of a CPM to dataset shifts described in terms of differences in patient populations, measurement, and missing data facilitates explaining the operating characteristics of the CPM.

## 6. Conclusion

In this manuscript we surveyed dataset shifts specifically connecting the ideas in statistics and epidemiology. We believe that unifying concepts in clinical research and ML provides a standardized framework on generalizability of CPMs. An important distinction we would emphasize is the difference between observed data differences and conceptual differences in data generating processes and mechanisms. This is similar to the distinction between population and sample characteristics that plague language in the statistical sciences. As an example, the phrase "the data is normally distributed" could describe the data generating mechanism or properties of the observed data. We prefer to define dataset shift as it relates to the processes underlying the data generation in lieu of the sample characteristics. Particularly, absence of empirical differences between datasets does not necessarily rule out one or more kinds of dataset shifts. Our proposed framework enables characterization of datasets in terms of ways to introduce shifts and therefore, assess the potential for dataset shifts. In turn, characterizing datasets in terms of factors that affect generalizability of CPMs can allow an explanation of failure modes of CPMs and their operating characteristics.

## Author Contributions

SV and BC conceived the article. BW and SV wrote the first draft. BW developed simulations. All authors agree to be accountable for the content of the work, revised the manuscript for critical content, and approved submission of the manuscript.

## Conflict of Interest

The authors declare that the research was conducted in the absence of any commercial or financial relationships that could be construed as a potential conflict of interest.

## Publisher's Note

All claims expressed in this article are solely those of the authors and do not necessarily represent those of their affiliated organizations, or those of the publisher, the editors and the reviewers. Any product that may be evaluated in this article, or claim that may be made by its manufacturer, is not guaranteed or endorsed by the publisher.

## References

[B1] Adebayo J. Gilmer J. Muelly M. Goodfellow I. Hardt M. Kim B. (2018). “Sanity checks for saliency maps,” in Proceedings of the 32nd International Conference on Neural Information Processing Systems, NIPS'18 (Red Hook, NY: Curran Associates Inc.), 9525–9536.

[B2] Altman D. G. Bland J. M. (1998). Generalisation and extrapolation. BMJ 317, 409–410. 10.1136/bmj.317.7155.409 9694763 PMC1113677

[B3] Altman D. G. Vergouwe Y. Royston P. Moons K. G. M. (2009). Prognosis and prognostic research: validating a prognostic model. BMJ 338. 10.1136/bmj.b605 19477892

[B4] Caffo B. Diener-West M. Punjabi N. M. Samet J. (2010). A novel approach to prediction of mild obstructive sleep disordered breathing in a population-based sample: the sleep heart health study. Sleep 33, 1641–1648. 10.1093/sleep/33.12.1641 21120126 PMC2982734

[B5] Copas J. B. (1983). Plotting p against x. J. R. Stat. Soc. C 32, 25–31. 10.2307/2348040

[B6] Damen J. A. A. G. Debray T. P. A. Pajouheshnia R. Reitsma J. B. Scholten R. J. P. M. Moons K. G. M.. (2019). Empirical evidence of the impact of study characteristics on the performance of prediction models: a meta-epidemiological study. BMJ Open 9, 26160. 10.1136/bmjopen-2018-026160 30940759 PMC6500242

[B7] Fügener A. Grahl J. Gupta A. Ketter W. (2021). Will humans-in-the-loop become borgs? merits and pitfalls of working with ai. Manag. Inf. Syst. Q. 45, 30. 10.25300/MISQ/2021/16553

[B8] Ghassemi M. Oakden-Rayner L. Beam A. L. (2021). The false hope of current approaches to explainable artificial intelligence in health care. Lancet Digital Health 3, e745–750. 10.1016/S2589-7500(21)00208-9 34711379

[B9] Hemingway H. Croft P. Perel P. Hayden J. A. Abrams K. Timmis A.. (2013). Prognosis research strategy (progress) 1: a framework for researching clinical outcomes. BMJ 346, e5595. 10.1136/bmj.e5595 23386360 PMC3565687

[B10] Hernán M. A. Cole S. R. (2009). Invited commentary: causal diagrams and measurement bias. Am. J. Epidemiol. 170, 959–962. 10.1093/aje/kwp293 19755635 PMC2765368

[B11] Hernán M. A. Hernández-Díaz S. Robins J. M. (2004). A structural approach to selection bias. Epidemiology 15, 615–625. 10.1097/01.ede.0000135174.63482.43 15308962

[B12] Justice A. C. Covinsky K. E. Berlin J. A. (1999). Assessing the generalizability of prognostic information. Ann. Internal Med. 130, 515–524. 10.7326/0003-4819-130-6-199903160-00016 10075620

[B13] Khudyakov P. Gorfine M. Zucker D. Spiegelman D. (2015). The impact of covariate measurement error on risk prediction. Stat. Med. 34, 2353–2367. 10.1002/sim.6498 25865315 PMC4480422

[B14] Kull M. Flach P. (2014). “Patterns of dataset shift,” in First International Workshop on Learning over Multiple Contexts (Nancy).

[B15] Luijken K. Groenwold R. H. H. Van Calster B. Steyerberg E. W. van Smeden M. (2019). Impact of predictor measurement heterogeneity across settings on the performance of prediction models: a measurement error perspective. Stat. Med. 38, 3444–3459. 10.1002/sim.8183 31148207 PMC6619392

[B16] Luijken K. Wynants L. van Smeden M. Van Calster B. Steyerberg E. W. Groenwold R. H.. (2020). Changing predictor measurement procedures affected the performance of prediction models in clinical examples. J. Clin. Epidemiol. 119, 7–18. 10.1016/j.jclinepi.2019.11.001 31706963

[B17] Moons K. G. Altman D. G. Reitsma J. B. Ioannidis J. P. Macaskill P. Steyerberg E. W.. (2015). Transparent reporting of a multivariable prediction model for individual prognosis or diagnosis (tripod): explanation and elaboration. Ann. Internal Med. 162, W1–W73. 10.7326/M14-0698 25560730

[B18] Moons K. G. M. Altman D. G. Vergouwe Y. Royston P. (2009a). Prognosis and prognostic research: application and impact of prognostic models in clinical practice. BMJ 338, b606. 10.1136/bmj.b606 19502216

[B19] Moons K. G. M. Kengne A. P. Grobbee D. E. Royston P. Vergouwe Y. Altman D. G.. (2012). Risk prediction models: Ii. external validation, model updating, and impact assessment. Heart 98, 691–698. 10.1136/heartjnl-2011-301247 22397946

[B20] Moons K. G. M. Royston P. Vergouwe Y. Grobbee D. E. Altman D. G. (2009b). Prognosis and prognostic research: what, why, and how? BMJ 338, b375. 10.1136/bmj.b375 19237405

[B21] Moreno-Torres J. G. Raeder T. Alaiz-Rodríguez R. Chawla N. V. Herrera F. (2012). A unifying view on dataset shift in classification. Pattern Recognit. 45, 521–530. 10.1016/j.patcog.2011.06.019

[B22] Oudega R. Hoes A. W. Moons K. G. (2005). The wells rule does not adequately rule out deep venous thrombosis in primary care patients. Ann. Internal Med. 143, 100–107. 10.7326/0003-4819-143-2-200507190-00008 16027451

[B23] Pajouheshnia R. van Smeden M. Peelen L. Groenwold R. (2019). How variation in predictor measurement affects the discriminative ability and transportability of a prediction model. J. Clin. Epidemiol. 105, 136–141. 10.1016/j.jclinepi.2018.09.001 30223065

[B24] Pearl J. (1995). Causal diagrams for empirical research. Biometrika 82, 669–688. 10.1093/biomet/82.4.669

[B25] Pearl J. Bareinboim E. (2011). “Transportability of causal and statistical relations: a formal approach,” in Proceedings of the Twenty-Fifth AAAI Conference on Artificial Intelligence, AAAI'11 (San Francisco, CA: AAAI Press), 247–254.

[B26] Riley R. D. Ensor J. Snell K. I. E. Harrell F. E. Martin G. P. Reitsma J. B.. (2020). Calculating the sample size required for developing a clinical prediction model. BMJ 368, m441. 10.1136/bmj.m441 32188600

[B27] Roberts M. Driggs D. Thorpe M. Gilbey J. Yeung M. Ursprung S.. (2021). Common pitfalls and recommendations for using machine learning to detect and prognosticate for covid-19 using chest radiographs and ct scans. Nat. Mach. Intell. 3, 199–217. 10.1038/s42256-021-00307-0

[B28] Rosella L. C. Corey P. Stukel T. A. Mustard C. Hux J. Manuel D. G. (2012). The influence of measurement error on calibration, discrimination, and overall estimation of a risk prediction model. Populat. Health Metr. 10, 20. 10.1186/1478-7954-10-20 23113916 PMC3545925

[B29] Royston P. Altman D. G. (2013). External validation of a Cox prognostic model: principles and methods. BMC Med. Res. Methodol. 13, 33. 10.1186/1471-2288-13-33 23496923 PMC3667097

[B30] Royston P. Moons K. G. M. Altman D. G. Vergouwe Y. (2009). Prognosis and prognostic research: developing a prognostic model. BMJ 338, b604. 10.1136/bmj.b604 19336487

[B31] Singh H. Singh R. Mhasawade V. Chunara R. (2019). “Fair predictors under distribution shift,” in NeurIPS Workshop on Fair ML for Health (Vancouver, BC).

[B32] Spiegelhalter D. J. (1986). Probabilistic prediction in patient management and clinical trials. Stat. Med. 5, 421–433. 10.1002/sim.4780050506 3786996

[B33] Steyerberg E. W. (2009). Clinical Prediction Models' A Practical Approach to Development, Validation and Updating. New York, NY: Springer. 10.1007/978-0-387-77244-8

[B34] Storkey A. (2008). “1-when training and test sets are different: characterizing learning transfer,” in Dataset Shift in Machine Learning, eds J. Quionero-Candela, M. Sugiyama, A. Schwaighofer, and N. D. Lawrence (Cambridge, MA: The MIT Press), 3–28.

[B35] Subbaswamy A. Adams R. Saria S. (2021). “Evaluating model robustness and stability to dataset shift,” in Proceedings of The 24th International Conference on Artificial Intelligence and Statistics, volume 130 of Proceedings of Machine Learning Research, eds A. Banerjee and K. Fukumizu (PMLR), 2611–2619.

[B36] Tonekaboni S. Joshi S. McCradden M. D. Goldenberg A. (2019). “What clinicians want: contextualizing explainable machine learning for clinical end use,” in Proceedings of the 4th Machine Learning for Healthcare Conference, Volume 106 of Proceedings of Machine Learning Research, eds F. Doshi-Velez, J. Fackler, K. Jung, D. Kale, R. Ranganath, B. Wallace, and J. Wiens (Ann Arbor, MI: PMLR), 359–380.

[B37] Um H. Tixier F. Bermudez D. Deasy J. O. Young R. J. Veeraraghavan H. (2019). Impact of image preprocessing on the scanner dependence of multi-parametric MRI radiomic features and covariate shift in multi-institutional glioblastoma datasets. Phys. Med. Biol. 64, 165011. 10.1088/1361-6560/ab2f44 31272093

[B38] Wells P. S. Anderson D. R. Bormanis J. Guy F. Mitchell M. Gray L.. (1997). Value of assessment of pretest probability of deep-vein thrombosis in clinical management. Lancet 350, 1795–1798. 10.1016/S0140-6736(97)08140-3 9428249

[B39] Whiting P. F. Rutjes A. W. Westwood M. E. Mallett S. Deeks J. J. Reitsma J. B.. (2011). Quadas-2: a revised tool for the quality assessment of diagnostic accuracy studies. Ann. Internal Med. 155, 529–536. 10.7326/0003-4819-155-8-201110180-00009 22007046

[B40] Wolff R. F. Moons K. G. Riley R. D. Whiting P. F. Westwood M. Collins G. S.. (2019). Probast: a tool to assess the risk of bias and applicability of prediction model studies. Ann. Internal Med. 170, 51–58. 10.7326/M18-1376 30596875

[B41] Wynants L. Collins G. Van Calster B. (2017). Key steps and common pitfalls in developing and validating risk models. BJOG 124, 423–432. 10.1111/1471-0528.14170 27362778

[B42] Wynants L. Van Calster B. Collins G. S. Riley R. D. Heinze G. Schuit E.. (2020). Prediction models for diagnosis and prognosis of covid-19: systematic review and critical appraisal. BMJ 369, m1328. 10.1136/bmj.m1328 32265220 PMC7222643

